# Supplementary material for: Identification of photoperiod-induced specific miRNAs in the adrenal glands of Sunite sheep (Ovis aries)
Source: Front Vet Sci. 2022 Jul 22;9:888207. doi: 10.3389/fvets.2022.888207 (PMC9354845; doi:10.3389/fvets.2022.888207)
Supplement: Supplementary file 1 [file Data_Sheet_1.ZIP › Supplementary file/miRDeep2 file.zip!/miRDeep2 file/Novel_Structure/LP42A2/Novel_219.pdf]

Provisional ID : NC_019467.2_18367
Score total : 1.6
Score for star read(s) : -1.3
Score for read counts : 0
Score for mfe : 1.3
Score for randfold : 1.6
Score for cons. seed :
Total read count : 3235
Mature read count : 3235
Loop read count : 0
Star read count : 0

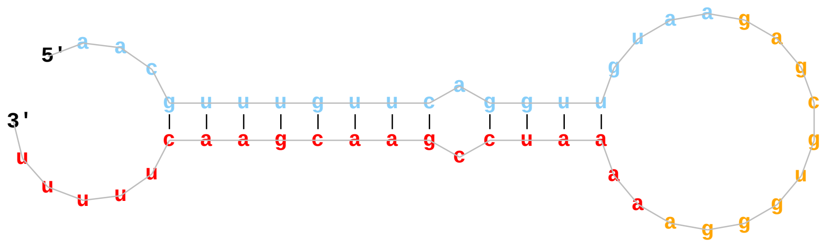

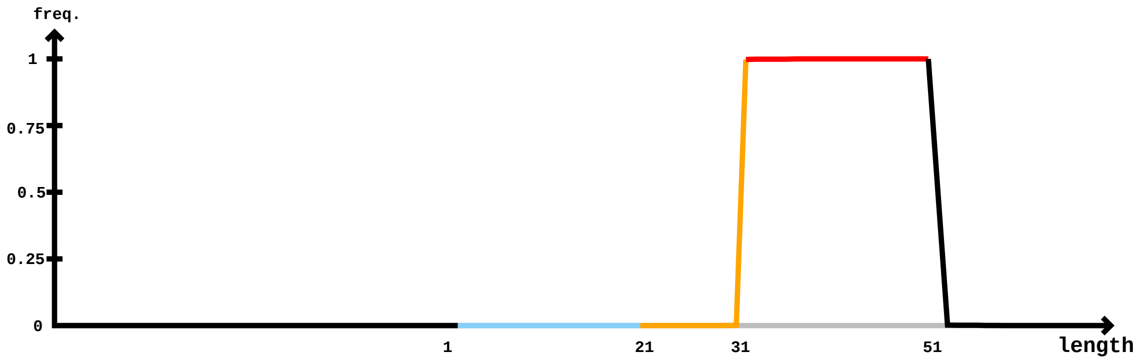

Star **Mature**

```
5'- uggguucuguccuagguaaugguaguacugguuggccaaaacguuuguucagguuguaagagcgugggaaaaauccgaacgaacuuuuuggccaagucaauaauuug   -3'   exp
    ..(((.(((.((........))))).)))((.((((((((((.((((((((.((((...............)))).)))))))).)))))))))).)).........    reads   mm   sample
    ...................................................................gaaaaaCccgaacgaacuuuu...................    1       1    seq
    ....................................................................aaaaaucUgaacgaacuuuuug.................    1       1    seq
    .....................................................................aaaaucUgaacgaacuuuuu..................    6       1    seq
    .....................................................................aaaauccgaacgaacuuuuu..................    3217    0    seq
    .....................................................................aaaauccAaacgaacuuuuu..................    3       1    seq
    .....................................................................aaaauccAaacgaacuuuuugg................    1       1    seq
    ......................................................................aaauccgaacgaacuuuuug.................    2       0    seq
    ......................................................................aaaucUgaacgaacuuuuugg................    1       1    seq
    ..........................................................................ccgaacgaacuuuuuggcca.............    3       0    seq
    ...........................................................................cgaacgaacuuuuuggccaag...........    1       0    seq
```
